# Supplementary material for: Comparison of RNA isolation methods on RNA-Seq: implications for differential expression and meta-analyses
Source: BMC Genomics. 2020 Mar 20;21:249. doi: 10.1186/s12864-020-6673-2 (PMC7082934; doi:10.1186/s12864-020-6673-2)
Supplement: Supplementary file 1 — Additional file 1: Figure S1. qPCR validation of representative differentially abundant transcripts. Figure S2. Properties of transcripts with differential abundance depending upon RNA isolation method. Figure S3. Properties of transcripts with differential abundance when RIN is included as a factor. Table S1. RNA concentrations and integrity (RIN) values. Table S2. Summary of mapping statistics. Table S3. Correlation coefficient (r) for log2 fold changes versus each factor. Table 4. Log2 fold-abundance changes versus each factor. [file 12864_2020_6673_MOESM1_ESM.pdf]

Supplementary Information

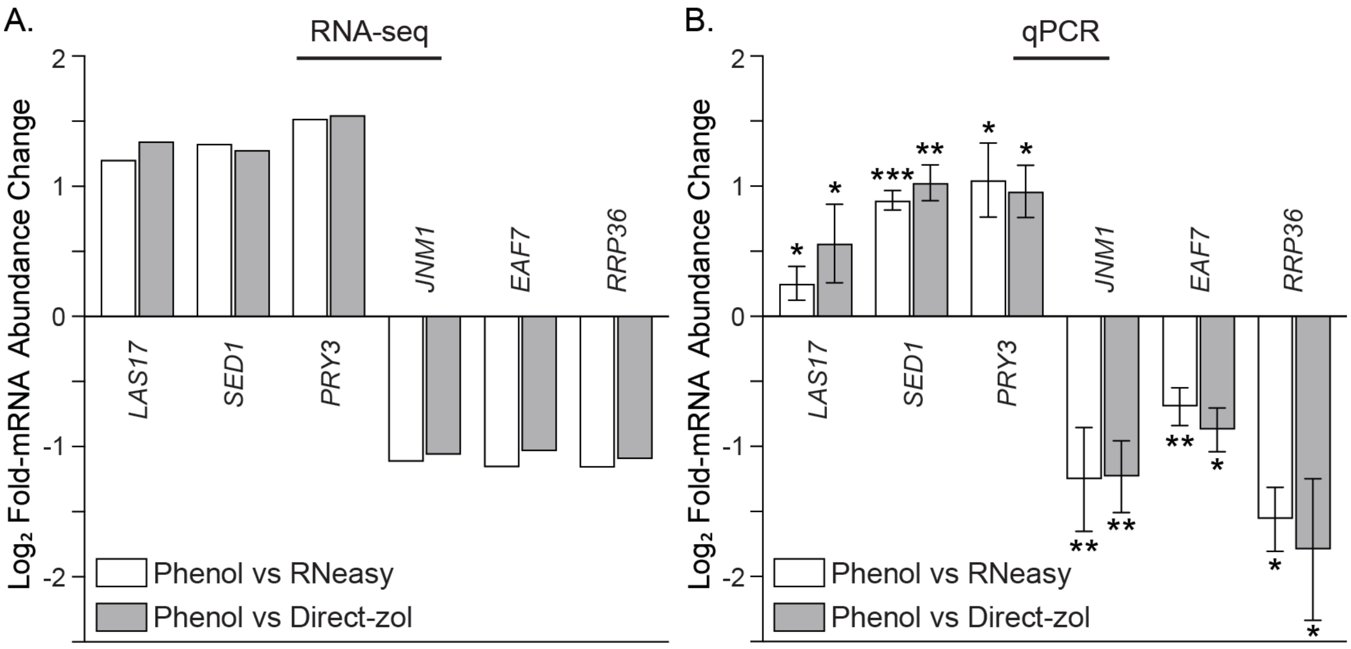

Supplementary Fig. 1. qPCR validation of representative differentially abundant transcripts. Representative transcripts with higher or lower transcript abundance in both of the Phenol vs. Kit comparisons as assessed by RNA-seq **(a)** were validated using real-time quantitative (q)PCR on the same identical RNA samples **(b)**. Error bars depict the standard deviation of the four biological replicates for the qPCR data. Asterisks represent means that are significantly different from zero (* $P < 0.05$, ** $P < 0.01$, *** $P < 0.001$, one-sample *t*-test).

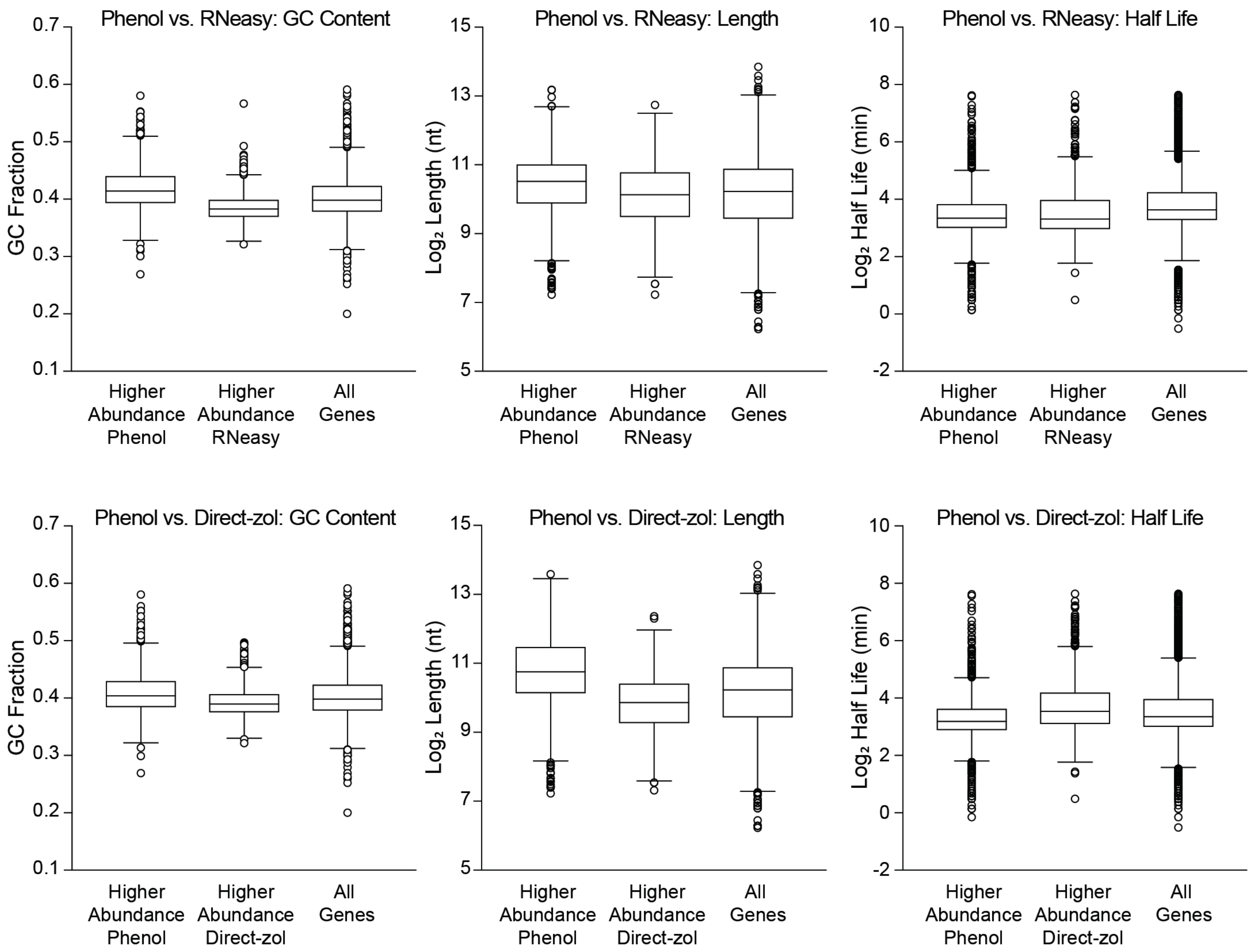

Supplementary Fig. 2. Properties of transcripts with differential abundance depending upon RNA isolation method. Boxplots depict the GC content (left), transcript length (middle), and *in vivo* half-life (obtained from [1]) for all genes with significantly higher abundance relative to their comparison group (FDR < 0.01) and all genes that passed filtering thresholds for differential expression analysis. For the Phenol vs. RNeasy comparisons, mean differences were significant by Mann Whitney *U* test for GC content ($P < 1 \times 10^{-15}$) and length ($P < 1 \times 10^{-15}$). For the Phenol vs. Direct-zol comparisons, mean differences were significant by Mann Whitney *U* test for GC content ($P < 1 \times 10^{-15}$), length ($P < 1 \times 10^{-15}$), and half-life ($P < 1 \times 10^{-15}$).

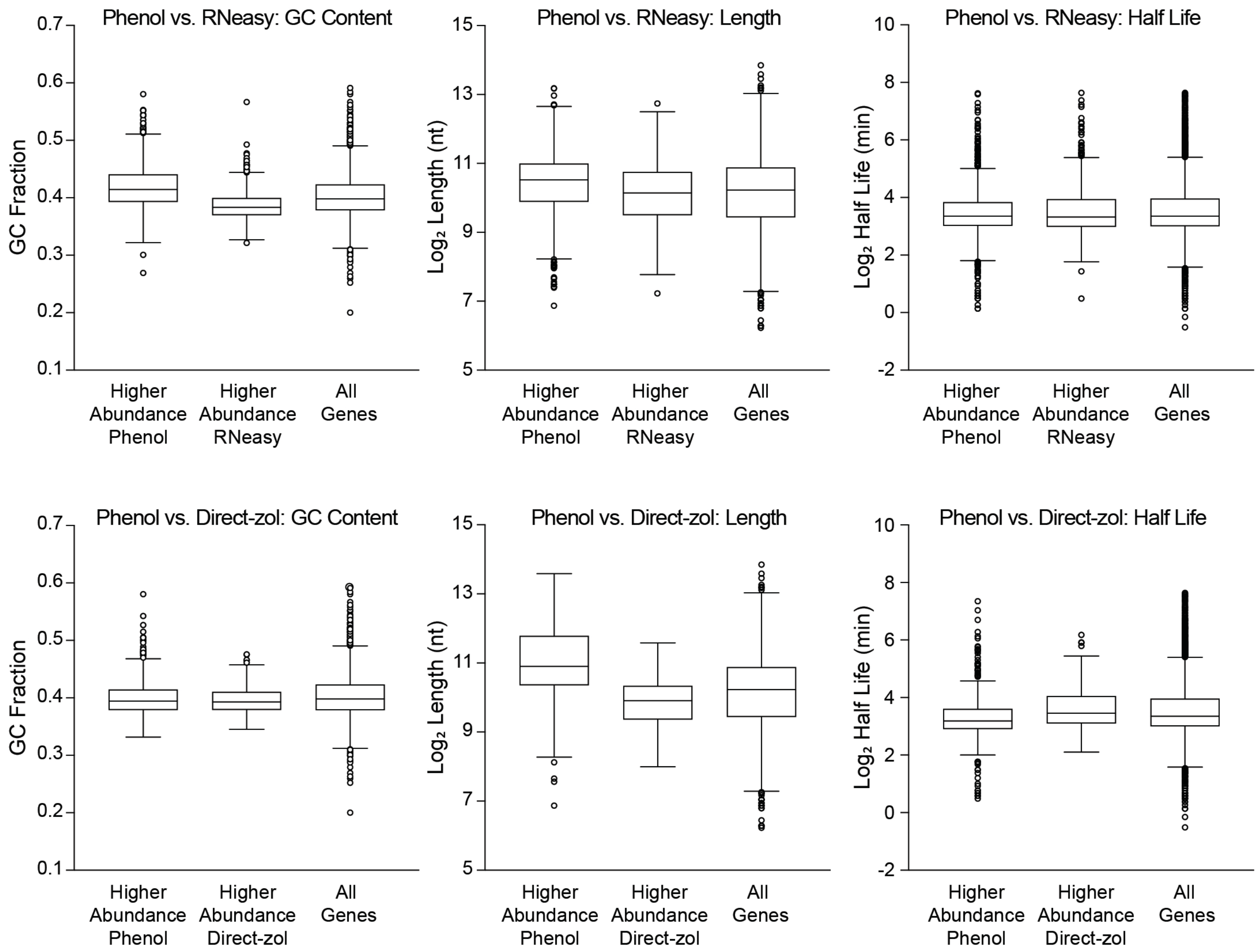

Supplementary Fig. 3. Properties of transcripts with differential abundance when RIN is included as a factor. Boxplots depict the GC content (left), transcript length (middle), and *in vivo* half-life (obtained from [1]) for all genes with significantly higher abundance relative to their comparison group (FDR < 0.01) with RIN included as a factor in the edgeR model, as well as all genes that passed filtering thresholds for differential expression analysis. For the Phenol vs. RNeasy comparisons, mean differences were significant by Mann Whitney *U* test for GC content ($P < 1 \times 10^{-15}$) and length ($P < 1 \times 10^{-15}$). For the Phenol vs. Direct-zol comparisons, mean differences were significant by Mann Whitney *U* test for length ($P < 1 \times 10^{-15}$), and half-life ($P = 4 \times 10^{-8}$).

Supplementary Table 1. RNA concentrations and integrity (RIN) values.

Sample ID	Concentration (ng / µl)	RIN
Phenol unstressed Rep1	1,100	9.9
Phenol unstressed Rep2	1,080	9.9
Phenol unstressed Rep3	960	9.9
Phenol unstressed Rep4	720	10
Phenol heat shock Rep1	610	10
Phenol heat shock Rep2	710	10
Phenol heat shock Rep3	770	10
Phenol heat shock Rep4	610	10
RNeasy unstressed Rep1	580	9.8
RNeasy unstressed Rep2	470	10
RNeasy unstressed Rep3	530	9.9
RNeasy unstressed Rep4	460	9.7
RNeasy heat shock Rep1	400	9.7
RNeasy heat shock Rep2	460	9.6
RNeasy heat shock Rep3	430	9.8
RNeasy heat shock Rep4	360	9.8
Direct-zol unstressed Rep1	590	9.4
Direct-zol unstressed Rep2	470	9.4
Direct-zol unstressed Rep3	620	9.5
Direct-zol unstressed Rep4	330	9.3
Direct-zol heat shock Rep1	260	9.0
Direct-zol heat shock Rep2	260	9.4
Direct-zol heat shock Rep3	560	9.3
Direct-zol heat shock Rep4	320	9.4

Supplementary Table 2. Summary of mapping statistics.

Sample ID	Input Reads[1]	% Uniquely Mapped	% Mapped
Phenol unstressed Rep1	13,270,392	83.58	96.21
Phenol unstressed Rep2	12,695,531	83.88	95.76
Phenol unstressed Rep3	12,856,634	85.13	96.32
Phenol unstressed Rep4	15,327,828	84.20	95.97
Phenol heat shock Rep1	10,900,974	86.5	96.57
Phenol heat shock Rep2	13,452,555	86.47	96.83
Phenol heat shock Rep3	14,946,326	87.32	96.82
Phenol heat shock Rep4	19,514,083	87.19	96.89
RNeasy unstressed Rep1	16,773,488	78.47	95.77
RNeasy unstressed Rep2	18,735,607	81.06	95.89
RNeasy unstressed Rep3	13,006,805	81.41	95.57
RNeasy unstressed Rep4	20,186,658	80.53	95.38
RNeasy heat shock Rep1	12,162,093	81.63	95.76
RNeasy heat shock Rep2	13,405,344	82.18	96.3
RNeasy heat shock Rep3	16,331,674	83.91	95.62
RNeasy heat shock Rep4	22,683,053	80.05	95.87
Direct-zol unstressed Rep1	16,815,144	86.38	96.91
Direct-zol unstressed Rep2	17,016,270	87.04	96.95
Direct-zol unstressed Rep3	11,598,964	85.87	96.73
Direct-zol unstressed Rep4	14,150,816	87.43	96.83
Direct-zol heat shock Rep1	14,678,584	88.02	96.72
Direct-zol heat shock Rep2	16,124,462	88.79	97.27
Direct-zol heat shock Rep3	16,753,180	87.92	96.89
Direct-zol heat shock Rep4	21,019,701	87.75	97.14

[1] Only reads surviving trimming were used as the input for STAR mapping.

Supplementary Table 3. Correlation coefficient (*r*) for $\log_2$ fold-abundance changes versus each factor.

Comparison[1]	Fraction GC	Transcript Length	Transcript Half Life[2]
Phenol versus RNeasy	0.32	0.06	-0.02
Phenol versus Direct-zol	0.14	0.23	-0.14
Phenol versus RNeasy: RIN	0.30	0.06	-0.01
Phenol versus Direct-zol: RIN	0.02	0.18	-0.03

[1] Comparisons were done either with or without RIN included as a factor in edgeR.
[2] Half-life estimates from Neymotin et al. [1].

Supplementary Table 4. Log$_2$ fold-abundance changes versus each factor.

Phenol vs. RNeasy				
Gene[1]	Log$_2$ Fold-Change	Fraction GC	Transcript Length	Transcript Half Life[2]
YBR116C	2.13	0.40	528	10.4
YLR342W-A	1.91	0.31	174	10.4
YNL194C	1.87	0.44	906	9.9
YPR192W	1.66	0.51	918	31.9
YJR146W	1.59	0.48	354	15.8
YDL078C	0.00	0.44	1032	17.1
YOR018W	0.00	0.39	2514	6.3
YHL031C	0.00	0.46	672	9.8
YGR109W-B	0.00	0.41	4644	2.3
YKR038C	0.00	0.44	1161	12.4
YKL082C	-1.16	0.38	1305	5.1
YNL136W	-1.16	0.38	1278	7
YOR287C	-1.16	0.37	903	10
YER038W-A	-1.16	0.44	381	2
YMR299C	-1.17	0.36	939	38.3
Phenol vs. Direct-zol				
Gene	Log$_2$ Fold-Change	Fraction GC	Transcript Length	Transcript Half Life[2]
YER190W	5.25	0.46	5046	6.9
YPL060C-A	2.29	0.33	3315	11.7
YDR535C	2.01	0.42	501	8.5
YNL194C	1.89	0.44	906	9.9
YPR202W	1.89	0.38	717	13.6
YLR397C	0.00	0.42	2343	9.5
YGR232W	0.00	0.39	687	43.2
YOR313C	0.00	0.36	1017	8.5
YOR147W	0.00	0.35	1869	14.2
YER045C	0.00	0.41	1470	6.8
YDR397C	-1.21	0.44	441	20
YPR200C	-1.23	0.38	393	29.1
YMR173W-A	-1.27	0.39	1185	21.4
YBL043W	-1.36	0.43	774	7.1
YKL065W-A	-1.78	0.31	222	34.4

[1] The genes selected had either the five highest, five lowest, or no abundance changes for the relevant Phenol vs. Kit comparisions.

[2] Half-life estimates from Neymotin et al. [1].

References

1. Neymotin B, Athanasiadou R, Gresham D. Determination of in vivo RNA kinetics using RATE-seq. RNA. 2014;20(10):1645-1652. doi: 10.1261/rna.045104.114 PMID: 25161313
